# Dysregulation of Multiple Signaling Neurodevelopmental Pathways during Embryogenesis: A Possible Cause of Autism Spectrum Disorder

**DOI:** 10.3390/cells10040958

**Published:** 2021-04-20

**Authors:** Jyoti Upadhyay, Jeevan Patra, Nidhi Tiwari, Nilima Salankar, Mohd Nazam Ansari, Wasim Ahmad

**Affiliations:** 1 Department of Pharmaceutical Sciences, School of Health Sciences, University of Petroleum and Energy Studies, Energy Acre Campus Bidholi, Dehradun 248007, Uttarakhand, India; jupadhyay@ddn.upes.ac.in (J.U.); jpatra@ddn.upes.ac.in (J.P.); 2 Institute of Nuclear Medicine and Allied Sciences, Defence Research and Development Organisation, Delhi 110054, India; tiwarinidhi0893@gmail.com; 3 School of Computer Sciences, University of Petroleum and Energy Studies, Energy Acre Campus Bidholi, Dehradun 248007, Uttarakhand, India; nilima11123@gmail.com; 4 Department of Pharmacology & Toxicology, College of Pharmacy, Prince Sattam Bin Abdulaziz University, Al-Kharj 11942, Saudi Arabia; 5 Department of Pharmacy, Mohammed Al-Mana College for Medical Sciences, Dammam 34222, Saudi Arabia; wasimahmadansari@yahoo.com

**Keywords:** autism spectrum disorder, Asperger’s syndrome, neuropathological alterations, hedgehog signaling pathway

## Abstract

Understanding the autistic brain and the involvement of genetic, non-genetic, and numerous signaling pathways in the etiology and pathophysiology of autism spectrum disorder (ASD) is complex, as is evident from various studies. Apart from multiple developmental disorders of the brain, autistic subjects show a few characteristics like impairment in social communications related to repetitive, restricted, or stereotypical behavior, which suggests alterations in neuronal circuits caused by defects in various signaling pathways during embryogenesis. Most of the research studies on ASD subjects and genetic models revealed the involvement of mutated genes with alterations of numerous signaling pathways like Wnt, hedgehog, and Retinoic Acid (RA). Despite significant improvement in understanding the pathogenesis and etiology of ASD, there is an increasing awareness related to it as well as a need for more in-depth research because no effective therapy has been developed to address ASD symptoms. Therefore, identifying better therapeutic interventions like “novel drugs for ASD” and biomarkers for early detection and disease condition determination are required. This review article investigated various etiological factors as well as the signaling mechanisms and their alterations to understand ASD pathophysiology. It summarizes the mechanism of signaling pathways, their significance, and implications for ASD.

## 1. Introduction

Autism Spectrum Disorder (ASD) is a chronic heterogeneous neurodevelopmental disorder characterized by impairment in social communications, related to restricted, repetitive, or stereotypical behavior [1]. It is the principal cause of disability among children less than 5 years of age suffering from mental disorders. It is more than conduct disorder and ADHD (Attention Deficit Hyperactivity Disorder) together because it persists throughout the life span of children suffering from this disease [2]. It is a great challenge for all developing countries, especially India, because of the severity of this disorder and its influence on the affected children and their families including the economic burden which imposes on the parents coupled with lack of knowledge about the disorder [3]. A lack of knowledge and awareness, wrong diagnosis, and inclusion of autism disorder under the normal classification of intellectual disability, language, or speech disorders are most commonly observed [4]. Some previous research studies suggested that early detection and intervention can improve language and speech abilities as well as reduce cognitive decline and behavior in affected children [5]. They also supported the idea that ASD can be diagnosed in the first two years after birth [6]; however, several studies report that a substantial proportion of affected children are not diagnosed until they are school-aged [7].

Worldwide data collected by survey analysis and routine monitoring structures show that several countries since the 1990s have identified an estimated rise in the prevalence of ASD from 0.7 to 1.0% [7]. The Centers for Disease Control and Prevention (CDC) in the United States conducted a study and reported that, from 11 sites, 1 in 54 children suffer from ASD, indicating an elevation in cases over the past two decades [8]. ASD prevalence in Asian countries has also been estimated, and it was found that in India, 1.7 to 2 million individuals are affected with ASD [9,10]. These rises in the number of ASD cases in the Indian population have increased the need to consider associated risk factors as well as to discover early detection techniques and therapeutic interventions.

Autism disorder was first identified in 1943 by Kanner in 11 children with similar symptoms like impaired speech and language, obsessive behavior, and deficits in social cognition. An epidemiological study on autism was conducted 23 years afterwards and estimated a prevalence rate of 4.5 per 10,000 persons. This estimated ratio was drastically increased to 1 in 59 persons, with males being diagnosed three times more than females [11]. This elevation in prevalence is the result of an increase in knowledge and awareness and an advancement in the Diagnostic and Statistical Manual of Mental Disorder (DSM) standards, which covers a broad range of disorders [12].

The etiology of ASD remains a topic of debate: Its origin, genetic and environmental factors, and their interplay form a range of cognitive, behavioral, and developmental features observed in affected individuals. The main aim of this review is to identify the etiological factors and pathogenesis and to characterize the methodological features together with recent therapeutic interventions. Unlocking the etiology of these multifaceted developmental disorders requires a collaborative research discipline including health care professionals, environmental experts, geneticists, bioinformaticians, and computer scientists.

## 2. Types of Autism Spectrum Disorder

ASD involves Asperger’s syndrome, autistic disorders, pervasive developmental disorders not otherwise specified (PDD-NOS), and Rett syndrome [13]. Asperger’s syndrome was identified in 1940 by pediatrician Hans Asperger. He observed symptoms of autism-like difficulty in communication and social interaction in boys with normal language development and intelligence. Many healthcare professionals have suggested that Asperger’s is a minor form of ASD and specified the term “high-functioning autism” to describe children affected with it [14].

The Diagnostic and Statistical Manual of Mental Disorders (DSM) is the standard guideline used by psychiatrists and physicians in the United States to diagnose any type of mental illnesses. In DSM-V diagnostic criteria for ASD, patients have persistent deficits in three areas of social interaction and communication: social-emotional reciprocity; non-verbal communicative behaviors; and maintaining, understanding, developing relationships. It also includes four types of restricted and repetitive behaviors: stereotypical repetitive motor movements, insistence or sameness, highly restricted or fixated interest, and hyper- or hypo-reactivity to sensory signals [15].

Without a diagnosis, PDD-NOS in children shows manifestations like restricted communications (verbal and non-verbal), social, and stereotypical behavior. Some epidemiological studies suggested that PDD-NOS are twice as common as autism [16]. The diagnostic features associated with PDD-NOS are (i) onset of this disease after 3 years of age, (ii) atypical symptoms, and (iii) Fewer than six criteria and their subthresholds [7,17]. A research study showed that children suffering from PDD-NOS were initially diagnosed with Attention Deficit Hyperactivity Disorder (ADHD) and that they did not differ from ADHD children with respect to all the symptoms of ASD, attention difficulties, or general psychopathology. No method has yet been established for differentiating ADHD and PDD-NOS [18]. Table 1 indicates the three severity levels of ASD.

The predictive etiology of ASD characterized by deficits in social communication and interaction as well as repetitive behavior is very complex and has not yet been established. Several factors including environmental, genetic, and epigenetic are found to be associated with this disorder. Important information has been obtained from the characterization of candidate genes through association and case-controlled studies and whole-genome sequencing involving genomic hybridization. Mechanistic studies involving epimutations, genomic imprinting, and methylation have been identified [19].

## 3. Etiology and Pathophysiology of ASD

Several factors are thought to be involved in the etiology of ASD. Non-specific signs like developmental delay, complications during pregnancy, dysmorphic features like an increase in head size indicate that ASD is a neuropsychiatric disease. Genetic and environmental factors play a key role in causing ASD. Some modeling studies reported that the interaction of several genes accounts for the underlying genetic complexity [20]. Thalidomide-associated embryopathy and antiepileptic drugs taken during pregnancy are the primary evidence of environmental factors associated with the etiology of ASD [21,22]. Some postmortem investigations using magnetic resonance imaging (MRI) identified an abundance of white matter and some structural impairment in cell alignment and density, especially in the limbic system [23]. Atypical stimulation of the amygdala and associated structures are analyzed by functional imaging techniques in response to social stimuli in ASD-affected children [24,25].

### 3.1. Genetic Factors

Some studies reported that siblings of autism-affected offspring have higher chances of having autism [13,26] and twin studies also reflected strong evidence for inheritance [23]. There is a broad range of phenotypes, but in the case of genetically homogenous autistic offspring they present with less phenotypic heterogeneity [27]. Studies of ASD on human and animal models [28] revealed rare variant mutations and de novo copy number mutations [29,30,31], which caused abnormal alleles in the affected person or close ancestors, thus affecting neuroanatomical and behavioral characters [32]. These relevant studies show a gene dysregulation mechanism involved in synaptic function [33]. Banerjee et al., studied the transduction mechanism involved in synapse formation in ASD and observed impairment in the structure of various transmembrane and protein scaffoldings involved in the synapse as well as dysregulation of the gene in the signal transduction mechanism that can be the major cause of genetic abnormalities in the etiology of ASD [34]. However, the cause of genetic mutation in ASD was discovered by how numerous genes interact in the affected person, together with epigenetic factors and environmental toxicants. Genetic factors involving identifiable diagnosable medical condition, cytogenetic problems and single gene defect comprises 25% of ASD cases [35,36,37]. Mutation in mitochondrial DNA is also a contributing factor because it leads to mitochondrial metabolism dysfunction [38]. Research studies suggested that the oligogenic mode of inheritance caused by hypomorphic multiple-gene alterations is one of the genetic causes behind intellectual disability and ASD. Another study showing whole exome sequencing (WES) of ASD patients suggested that no single gene was responsible for ASD-related risk factors; instead, it postulated the involvement of risk variants distributed across hundreds of genes [39]. Table 2 represents the list of genes involved in ASD risk. Several of them are found to be associated with ASD that acts as a major player in synaptic development and function, like *NRXNI* (neurexin 1 α), *CNTNAP2* (contactin-associated protein-like), *NLGN4* (neuroligin), *SHANK1, SHANK3* (SH3 and multiple ankyrin repeat domains 3). Research on postmortem analyses of ASD patients reported that a certain number of neurons were reduced in the amygdala, cerebellum, and gyrus (fusiform) and that there were signs of neuroinflammation [40]. Oblak et al., also reported in autistic children a decline in the serotonin transporter density in the deeper tissues of the fusiform gyrus [41].

### 3.2. Neuropathological and Neurotransmitter Level Abnormalities in the ASD Brain

Several works in the literature suggested that neuropathological alterations occur in certain areas of the brain, including the cerebellum, cortex, and limbic system. Some parts of these structures, instead of the entire structure, are found to be the most exaggerated, such as the fusiform face area and amygdala nuclei. Cortical organization impairment characterized by narrower and repeated minicolumns as well as the overgrowth of the frontal region of the brain during early development, affects connectivity. The presence of excess white-matter neurons, focal cortical dysplasia, fewer GABAergic Purkinje cells in the cerebellum, cytoarchitectonic laminar alterations, and other events that cause anomalies in nerve transmission are the abnormalities that can be traced during the growth and developmental period [56,57].

#### 3.2.1. Olivocerebellar Impairment

##### Cerebellum Impairment in ASD and Decrease in Purkinje Cells

Impairment in the cerebellum and a decrease in the number of Purkinje cells occur in this type of neuropathological abnormality. The cerebellum functions as an “error correction system” that regulates motor function, coordination, and body balance involving head and neck movement and spatiotemporal positions. Proprioceptive sensory signals from the muscles and auditory, visual and somatosensory signals are received by the cerebellum and sent through the thalamus (lateral and anterior) and red nucleus to the motor cortex. The cerebellum performs a major function in cognition. Any injury to this region (stroke or cerebellar lesions) were found to be associated with abnormality in communication, verbal ability, high-order brain functions, and cognition-related activities [57]. Some studies reported that in 156 ASD patients, cerebellar lesions cause altered cognitive patterns associated with language processing, sequence information, visuospatial ability and memory, executive function, and attention [58]. The most attractive area of the cerebellum is the posterolateral hemispheric region having Crus I and Crus II, which receive a large amount of frontopontine projections [59] and have been reported to have numerous deficit Purkinje cells [60].

##### Cholinergic Receptor Changes in the Cerebellum

Martin-Ruiz et al., performed the molecular analysis of cholinergic receptor expression in ASD. They reported a 40–50% decline in cholinergic nicotinic receptor types (𝛼𝛼3 and 4, 𝛽𝛽2) determined by using the agonist epibatidine in a Purkinje cell. The granule cell and molecular covering of the cerebellum observed in postmortem autism cases compared with reference control [61]. Another ligand 𝛼𝛼-bungarotoxin was used to measure cholinergic nicotinic receptor type 𝛼𝛼7 and a three-fold increase in the level of this receptor was reported. The 𝛼𝛼7 receptor is located on the cell surface of inhibitory GABA neurons and stimulation of these receptors causes GABA release, thus playing an important role in restoring inhibitory neuronal activity. The use of anticholinergic and some antidepressant drugs helps to lessen autistic symptoms and is suggested to be a novel drug treatment for ASD [62]. However, there is insufficient evidence to prove the effect of GABA modulators because almost all the evidence is focused on children, and there is no specific study designed for adults. Hence, future research from both clinical and preclinical studies will shade more light on the therapeutic effectiveness of GABA modulators in ASD [63].

##### Inferior Olivary Complex (IOC) Pathology

The IOC is the part of the brainstem intimately associated with the cerebellum, supplying directly olivocerebellar fibers to the Purkinje cell dendrites in the cerebellum [64]. Postmortem studies in of an autistic brain analyzed the IOC association with the cerebellar hemisphere and found it to be irregularly small: some pale stained neurons with no loss of cell function [65]. There were also age-related variations in olivary neuronal size and distribution in the nuclei, such as small-size neurons in appropriate numbers and irregular neuronal distribution along the periphery and edge of the principal olive loop in adults (above 22 years of age) [66]. However, in an autistic child, the principal olive neurons were adequate in number, significantly bigger than the control, and had abnormal neuronal distribution along the olivary ribbon edges [66]. Bailey et al., [67] investigated six autism postmortem cases and observed a flattened medulla oblongata containing pyramids and IOC in one case and small pyramids in a large medulla in another. They also observed “breaks” in the area of the inferior olive in three cases, duplication in the area of the neuronal ectopia and olivary ribbon in four cases, and a small collection of neurons in cerebellar peduncles (inferior) in three cases [68].

##### Deep Cerebellar Nuclei (DCN) Pathology

The mechanism of DCN pathology is unclear in autism as the literature related to the quantitative study of nuclei (dentate nuclei, globose nuclei, fastigial nuclei, and emboliform nuclei) is not available. Bauman and Kemper’s observations indicated Purkinje cell reduction and atrophy of folia in the granular layer of the cerebellar hemisphere. Three small and pale deep-cerebellar nuclei were reduced in number, and a distorted dentate nucleus containing an adequate number of pale cells was also observed [66]. An in situ hybridization study using a 35S probe measured glutamic acid decarboxylase 65 type (GAD 65). It labeled two marked neuronal fibres within the dentate nuclei in the adult ASD population, which matched reference cases [67]. The GAD 65 was found to be present in two classes of dentate neurons, one of which is about 10 micrometers in diameter and another about 20 micrometers. The smaller neuronal population was presumed to be GABAergic neurons that innervate other dentate neurons, and in an animal model the larger one projected out of the cerebellum into the IOC [69]. The larger neuronal population contained a significantly lesser amount of GAD 65, suggesting the inhibitory role of IOC may have an impact on autism.

##### Brain Stem Impairment Pathology

A decrease in grey matter volume in the brainstem was observed in autistic subjects with no difference in white matter volume as observed by magnetic resonance imaging (MRI) in 22 non-mentally retarded boys with autism and 22 older, gender-matched controls [70]. This study correlated the observations of the oral sensory sensitivity and brain stem grey-matter volume as determined by a sensory profile questionnaire (SPQ) [14,15]. In 2000, Rodier explained the abnormalities in female autistic subjects by showing the absence of a “band” of neuronal tissue in the brainstem that influences other related structures. He described the absence of the facial nucleus, which regulates the muscles of facial expression, and the relay of auditory transmission. The superior olive showed that these two structures are developed from the same neural tube of the embryo, demonstrating early developmental defect after four weeks [71].

#### 3.2.2. Limbic System Impairment

##### Hippocampus Pathology

Impairment in the hippocampus region was reported in early 1985 studies in the postmortem of autism patients. The histological examination of the brain demonstrated a Nissl-stained hippocampus with an elevated number of neurons per unit volume throughout the Cornu Ammonis (CA) and subicular subfields [65]. The flattened appearance of the hippocampus was observed in autistic patients as compared to control because of the high cell-packing density in the hilar CA4 subfield. Reduction in the pyramidal cell size in the hippocampus was also observed [66]. Dendritic arbors and decreased complexity in the CA1 and CA4 region was observed by Golgi analysis [72].

##### Entorhinal Cortex Pathology

The declarative memory formation in the brain is dependent upon the hippocampus and parahippocampus, and abnormalities in the parahippocampal gyrus and entorhinal cortex were observed in autism cases. Postmortem cases revealed that in the clear zone interior to the superficial membrane, the lamina dissecans was present in the adult autism subjects, whereas in control subjects it disappeared during childhood [65].

##### Amygdala Pathology

Elevations in the cell-packing density in specific amygdalar subregions were observed, which also included a 30–35% reduction in cell size in the central, cortical, and medial nuclei. Minor changes were observed in the basolateral complex [65]. Stereology and fractionator methodology were used throughout the whole amygdala and entire nuclear regions to establish baseline counts in 10 postmortem cases with neurological impairment. These results, when combined with the MRI studies, reported that the amygdala of autistic cases underwent abnormal growth and development postnatally and showed enlarged and reduced neuronal numbers [73,74].

##### Anterior Cingulate Cortex (ACC) Pathology

The anterior cingulate cortex (ACC) performs several functions involving execution, evaluation, cognitive function, and emotions [75]. Processing of information (sensory and multimodal) occurs through connections with the motor system and the parietal and prefrontal cortex, including the frontal eye fields [76]. Learning and problem-solving––as well as an emotional reaction to pain, motivation, task anticipation, social interaction, and control of social and emotional responses––are all functions performed by the anterior cingulate cortex [77,78]. It has also been assumed that the ACC plays a role in theory of mind because of its circuitry linkage with the temporoparietal junction and adjacent part of the frontal cortex [79]. This circuitry linkage is also found to be involved in joint attention, which is found to be absent in many autism patients [80]. Any impairment at the molecular, cellular, or chemical level of this region leads to communicative, behavioral and social deficits.

##### Posterior Cingulate Cortex (PCC) Pathology

The posterior cingulate cortex shows laminar impairment similar to ACC when observed in postmortem autism cases. Eight cases were examined via tissue block sections with Nissl stain and in one case there was an increased number of neurons in layer 1 and in the cell-packing density in layer III. An irregular distribution of large neurons in layer II and layer V, and a smaller neuron distribution in layer III were observed in another case of autism. The other four cases showed abnormalities in layer V and three cases showed similar findings to that of ACC: increased cell density of white matter neurons. This cortical impairment showed abnormal cortical development as well as neuronal migration to the cortical plate from the ventricular germinal zone that occurs between 8 and 22 weeks of gestation [81].

##### Default Network Defects Pathology

The default mode network (DMN) is active during cognitive processes and passive resting conditions and is found to be defective in autism patients. Such defects in the default mode network might be related to social deficit conditions in autistic subjects like theory of mind [82]. According to the Autism Diagnostic Observational Schedule (ADOS) and the Social Responsiveness Scale (SRS), a data-driven study of DMN subnetworks endorsed the hypothesis that the DMN subnetwork, which is connected to the anterior and frontal cingulate with the precuneus, leads to core deficits in ASD patients. Some studies described default networks that include the lateral parietal cortex, posterior cingulate cortex, medial prefrontal cortex, temporal lobe, retrosplenial cortex, and parahippocampal gyrus. These are strongly active when there is no task to be performed [83]. The functional magnetic resonance imaging (fMRI) studies reported that poor social interaction in ASD subjects was associated with weaker network connections between the PCC and frontal cortex. However restricted interest and repetitive behavior in ASD subjects were associated with stronger connectivity between the PCC and parahippocampal gyrus [83].

#### 3.2.3. Neocortical Pathology

##### Cortical Dendritic Impairment

Some researchers using the Golgi method found some alterations in the spine densities of the dendrites on projecting cortical neurons. They examined frontal, parietal, and temporal lobes and observed increased density of dendrite spines in layer II in three of the cortical regions of the lobes and in layer V of the cortex region of the temporal lobe. The higher density of spine dendrites was associated with a decrease in brain weight, which is commonly found in cognitively impaired ASD subjects. The authors speculated that increased spine densities could be caused by a failure to cull connections during the prenatal and postnatal periods, as shown by changes in the density of synapses (excitatory) to the lobes’ cortical neurons [56].

##### Abnormal Organization of Cortical Minicolumns Pathology

Alterations in cortical minicolumns in layer III of the temporal and prefrontal cortex in ASD was observed in some of the research studies [56]. Minicolumns are the modular organization of neurons that span all neocortical layers of the brain and serve to arrange neurons in a defined shape and space and possess similar properties [24]. Large minicolumns are thought to play an important role in generalization, whereas small minicolumns facilitate discrimination. A small neuropil space at the periphery of the minicolumns in autism subjects suggests that GABAergic innervation to the minicolumn neurons interferes with the processing and differentiation of signals [24].

##### Abnormality in Frontal Lobe Growth Pathology

Autism-affected children of about 2–3 years of age display behavior having abnormalities in attention, motor, social and sensory functions. It has been observed that developmental abnormalities in brain-growth features like an enlarged cerebrum, cerebellum, and limbic structure occur in autism subjects at about this age [56]. Abnormal growth of the cortical region, especially the frontal lobes of the cerebrum, has been hypothesized to adversely affect the development of synaptogenesis, circuit formation, and dendrite growth, which ultimately affects social interaction, language, emotion processing, and cognition [84].

### 3.3. Non-Genetic Factors Implicated in ASD

Other inflammatory conditions, gastrointestinal problems, environmental toxins, diet, and infections are found to be associated with ASD.

#### 3.3.1. Gastrointestinal Implications

Gastrointestinal abnormalities associated with ASD have received significant attention due to their evidence-based prevalence and associations with severe clinical symptoms. The most common GI symptoms include abdominal pain, diarrhea, and chronic constipation [85]. Gastroesophageal reflux disease (GERD), vomiting, bloody stools, and gaseousness are found to be elevated in ASD, which indicates signs of gastrointestinal inflammation like hyperplasia of the lymph node, complement activation, increased levels of proinflammatory cytokines, and intestine-related pathologies such as gastritis, esophagitis, and enterocolitis [85]. The gastrointestinal tract maintains homeostasis by working with the immune system to protect the body against foreign particles and microbes. The intestinal mucosa comes continuously in touch with a large number of foreign particles and microorganisms from the surroundings. The intestinal mucosal barrier is organized in such a way that it maintains the immune function of the mucosa and helps prevent inflammation. Apart from gut microbial flora, the response of the mucosal immune system regulates the T helper cell (Th2 vs. Th1) population responses. The intestinal mucosal epithelium represents MHC cells, both classical and non-classical, and causes activation of particular regulatory T cells; hence, it acts as non-professional antigen presenting cell (APC). The various components of our intestinal barrier consist of mucus production, innate immune response, epithelial cell integrity, and permeability. Any alterations to these components cause intestinal inflammatory diseases [86]. Several research studies reported GI infection in ASD subjects. The infection can alter intestinal permeability, permitting the entry of *E. coli* bacteria into the intestinal cells, impairing actin dynamics, modulating immune function, and leading to the disruption of tight junctions. This increase in intestinal permeability ultimately results in diarrhea [87].

#### 3.3.2. Immune System Imbalance in ASD 

Several studies supported the evidence that abnormalities of the immune system, such as the activation of microglial cells and neuroimmune-system-causing neuroinflammation are found in the cerebrospinal fluid and brain [88]. Alterations in the blood–brain barrier, increased cytokine levels and immune system dysregulation were also observed in ASD patients [89], as were immune system-associated problems in the GI tract and CNS. Maternal inflammation and autoimmune disorders of the family of ASD children are associated with immune system abnormalities in the offspring.

#### 3.3.3. Neuropeptides in ASD (VIP, BDNF, CGRP)

The presence of an increased number of neuropeptides like vasoactive intestinal peptide (VIP), brain-derived neurotrophic factor (BDNF), gene-related peptide (CGRP), and neurotrophin (NT4/5) in the blood samples of 60 neonates has opened many lines of ASD investigation [90]. The VIP glucagon secretin family contains pituitary adenylate cyclase-activating peptide (PACAP) and VIP, which performs various functions in the lungs, kidney and the cardiovascular, digestive and endocrine systems and is found to be involved in the development of neurons, astrocytes, and cerebrum. VIP is a neurotransmitter in the parasympathetic nervous system as well as a neuromodulator involved in the pathology of cluster headaches [91]. It functions during the stimulation of brainstem reflexes and is found to lower blood velocity in the middle portion of the cerebral artery. Both the PACAP and VIP are involved in the homeostatic mechanism of the immune system and thought to have an anti-inflammatory effect on the adaptive and innate immune responses, thereby promoting Th2 action and suppressing pro-inflammatory Th1 responses [92,93]. The Neurotrophic agent BDNF of the nerve growth family, along with other neurotrophins, acts on tyrosine kinase receptors with greater affinity and acts on the low-affinity p75 receptor. The BDNF at low concentrations can excite neurons in the cerebellum, cerebral cortex, and hippocampus. Neurotrophin NT4/5 and the BDNF were found to depolarize the neurons of the brain as common as glutamate (excitatory neurotransmitter) at thousand folds lower concentrations. The deficiency of the BDNF was found to be implicated in several psychiatric diseases and ASD in various animal models [94,95].

In sensory neurons, the CGRP is a neurotransmitter and neuromodulator that coexists with substance P, neurokinin A (NKA), and glutamate as an endogenous vasodilator peptide [96]. The CGRP increases the release of the BDNF from trigeminal ganglia, suggesting that the BDNF might be an endogenous mediator of nociception plasticity [93]. Brondino et al. [97] suggested that it may also be part of a biochemical continuum that essentially focuses on autistic characters in the general population. Further research studies with a large population size is required for a better understanding of the biological systems underlying individual variation in ASD characters in both non-clinical and clinical populations.

## 4. Impairment of Developmental Pathways

### 4.1. Wnt Protein and β-Catenin Signaling Pathways

**Figure 1:**
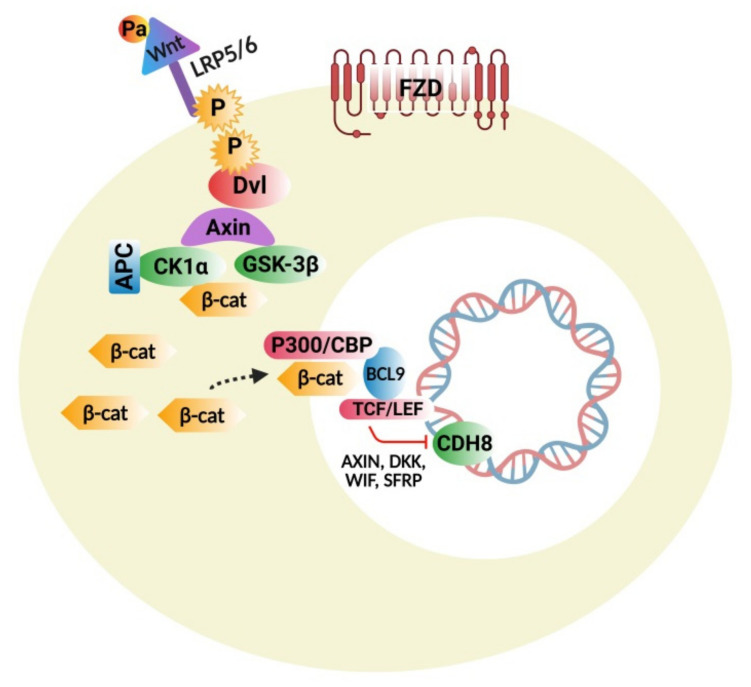
Wnt signaling pathway: Binding of Wnt to frizzled receptors (FZD) and LRP5/6; phosphorylation of LRP5/6 by GSK-3β; CK1α attracts the Dvl to the membrane and then inhibits the destruction complex; β-catenin in the cytoplasm is translocated to the nucleus, dislodging Groucho repressor and recruiting various BCL9 co-factors by binding to LEF/TCF. BCL9 and CBP/p300 permit the transcription of Wnt targeted genes, which are involved in cell differentiation, proliferation, and adhesion.

The Wnt protein family consists of several molecules that act as important regulators of stem-cell renewal, embryonic growth and development, cell proliferation, and tissue homeostasis [98]. This signaling cascade is divided mainly into two branches: β-catenin dependent canonical and non-canonical pathways, which are further classified into calcium dependent and a planar-cell polarity nerve tract. Figure 1 represents the Wnt/β-Catenin Signaling Pathway. Beta-catenin is an endogenous protein that is encoded by the gene CTNNB1, an adherent junction component bound to E-cadherin. The key components of the canonical pathway in humans consist of numerous Wnt ligands, low-density lipoprotein 5/6 co-receptors, frizzled receptors, and intracellular and extracellular modulators [99]. Wnt ligands are proteins rich in cysteine and have substantial post-translational modifications, including glycosylation and palmitoylation, which are important for biological actions [100].

#### 4.1.1. Wnt/β-Catenin Signaling Disturbances

Research studies on transgenic animal models show the importance of Wnt proteins in the normal growth and development of the CNS and the outcomes caused by any potential disorders [101]. The important gene mutated in ASD is CHD8 that encodes for ATP-associated chromatin remodeler peptide an important modulator of the Wnt pathway [102]. In neurons, the CHD8 gene works as a negative regulator of the Wnt protein, and mutations induced by the loss of function are related to the overexpression of Wnt-dependent genes [103]. Some in vivo studies reported that the CHD8 loss of function can stimulate more canonical Wnt signals causing macrocephaly (during the early stages of the development of the autistic brain) and induce ASD-like symptoms [104]. Frizzled class receptor 9 (FZD9) is important for the canonical pathway Wnt2 ligand. FZD9 gene mutation has been implicated in various ASD subjects [105]. Duplications and the loss of FZD9 receptor functions were found to be associated with ASD, causing a fine balance during brain development. In the mice model, administration of Wnt 2 causes overexpansion of the neurons of dopaminergic pathways in the midbrain resulting in repetitive behavior in ASD subjects [106].

#### 4.1.2. Wnt Pathway Modulators

In the past few years, the chemical Arsenal was found to modulate Wnt signal pathways, and monoclonal antibodies and some drugs directly inhibited the components of Wnt signaling pathways or block the interaction of proteins through downstream effectors like β-catenin and its transcription proteins. Examples include soluble decoy receptors that bind Wnt, antibodies that act against FZD receptors, and some protein disheveled (Dvl)-targeting small-drug inhibitors. Some of the non-specific pathway modulators were also isolated like tankyrase inhibitors, which damage Axin and suppress the canonical pathway of Wnt signaling [107]. The compound XAV939 was given prenatally to mice to cause ASD-like symptoms [108]. An porcupine enzyme catalyzes the palmitoylation of Wnt and activates this pathway. The porcupine antagonist LGK974 or WNT974 shows the potential effects in the treatment of solid tumors. It was found that compounds like TCF (T cell factor), CBP/p300 (CREB-cAMP response element) binding protein, and BCL9 (B-cell CLL/lymphoma 9 transcription co-activator) block the interaction of β-catenin with other co-activators or co-factors [107].

#### 4.1.3. Interconnections of Wnt Signaling with Developmental and Inflammatory Pathways

Wnt-signaling proteins modulate development-related pathways. Both Wnt and Hedgehog signaling influence each other at their transcriptional levels [109]. Cell-line studies demonstrated that Gli (Glioma-associated oncogene/transcription factor) promotes translocation of β-catenin at the nuclear level through E-cadherin and Snail [110]. Gli elevates Wnt signaling by regulating WNT5a and WNT2b genes [111]. Both of these signaling pathways share some common protein modulators like Glycogen synthase kinase 3β (GSK-3β), Casein kinase 1α (CK1α), Phosphatase and tensin homolog (*PTEN*), and p53. Together, they activate β-catenin and Gli signaling [112]. Embryonic developmental studies revealed the interaction of Wnt signaling with transforming growth factor TGF-β, retinoic acid signaling, and bone morphogenetic protein (BMP) [113,114,115]. Wnt signaling can also interact with cytokine signaling and nuclear factor kappa (NF-κB) in many ways to stimulate inflammatory responses [116].

Any therapeutic agent modulating ASD-related pathways may have beneficial effects on the Wnt signaling system. It is important to investigate only the several therapeutic agents targeting the Wnt signaling cascade to avoid the collateral effects on other pathways, like Imatinib the tyrosine kinase inhibitor that inhibits the phosphorylation of receptors, especially growth factor, and decreases the signal cascade of the Wnt pathway [117]. Therefore, it is essential to avoid the collateral effect of therapeutic agents on other pathways while acting on the Wnt signaling cascade. It is crucial to investigate Wnt transcriptional agents and explore further investigations into transcriptional agents, including β-catenin and other related cofactors like BCL9 (B-cell CLL/lymphoma 9 transcription co-activator) and T-cell factor with special reference to ASD. This study will be of significant relevance to future pharmacological therapeutic interventions and the customized design of ASD-modifying drugs.

### 4.2. Hedgehog Signaling Pathway

**Figure 2:**
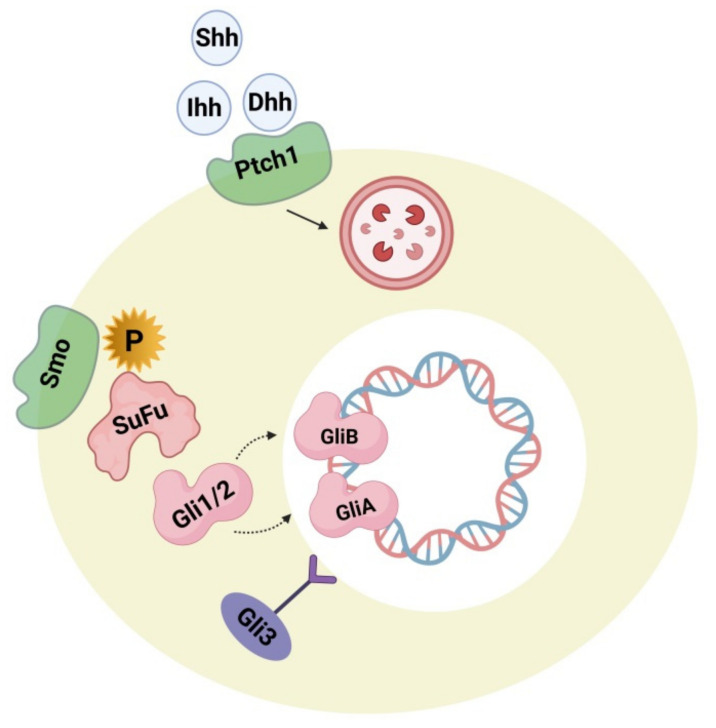
The hedgehog (hh) signaling pathway showing hh ligands (Shh, Dhh, Ihh) and their receptor Ptch 1. Upon binding with Ptch1, the pathway causes internalization, and Smo inhibition is released. After this, Smo is phosphorylated causing a cascade activation through downstream regulation, and Gli1/2 is processed into the activator forms GliA and GliB. After translocation of GliA into the nucleus, it stimulates target gene expression. The transcriptional repressor precursor Gli3 remains inactive.

Hedgehog plays a major role during embryogenesis by acting directly on neural development and the dorsoventral pattern. During adulthood, it exerts a role in neuron generation, phenotype determination of neurons, cell cycle, stem-cell maintenance, and apoptosis. These processes include canonical and non-canonical mechanisms [118]. Figure 2 represents the hedgehog signaling pathway, the impairment of which is lethal during embryogenesis, causing serious birth defects like cyclopia and holoencephaly. In the adult human brain, the hedgehog-signaling pathway is executed in a paracrine manner through sonic hedgehog ligand-secreting neurons in the ventral forebrain, substantia nigra, and septum as well as by ligand-activated glial cells in the sub-ventricular, ventricular, sub-granular zone and cortex [119]. Some research studies supported evidence that the sonic hedgehog-signaling pathways perform an important role in cortical neuron fate modulation, circuit establishment, and astrocyte-arbitrated synaptic plasticity [120]. Changes in the cellular response of hedgehog production and pattern can be observed in brain-related disorders like neoplasia, brain-cell injury, and psychiatric disorders [121]. Some studies reported the correlation of components and hedgehog signaling-pathway activation (e.g., Patched activation, ligand concentration, cellular localization) leads smoothly to an etiology of ASD. Research in this field investigated the associated link between hedgehog components in the autistic brain and their synergism with several confounding factors, including congenital mutations in the gene component of the hedgehog pathway, impairment of the oxidative stress defense system, and inborn cholesterol metabolic errors. High serum levels of sonic hedgehog proteins were detected in autistic subjects when compared with an age-matched control group. The severity of the condition was positively correlated with the serum level of sonic hedgehog proteins. In addition to this, it was also observed that, in autistic subjects, blood levels of hydrogen peroxide (H_2_O_2_), superoxide anion (O_2_^−^), and hydroxyl radicals (OH ^−^) were significantly higher. Thus it was suggested that increased oxidative stress induces the activation of a sonic hedgehog-dependent neuroprotection mechanism [121,122]. Other reports also contributed to the hypothesis that additional anti-oxidative pathway components like BCL2 (B-cell CLL/lymphoma 2 apoptosis regulator) apoptosis regulator, glutathione peroxidase, superoxide dismutase, and the BDNF (brain-derived neurotrophic factor) might cause some changes to the sonic hedgehog protein concentration [123]. Other studies determined an interface between autism phenotypes and the Indian hedgehog and Desert hedgehog proteins. Investigations have shown that the serum concentration of desert hedgehog is lowered in autistic subjects with no exact correlation with disease severity [124]. Indian hedgehog proteins in serum were elevated in autistic subjects more significantly if there was a positive correlation with the severity of the disease [125]. Hence it is suggested that hedgehog proteins along with oxidative stress components may be significant biomarkers for ASD.

During brain development, the hedgehog pathway is interrelated with several developmental and cell-survival mechanisms. Pathway activation can be implemented by both canonical (patched 1 mediated) and non-canonical processes. The non-canonical mechanism is mediated by several kinases like PKA (phosphokinase), GSK3-3β (Glycogen synthase kinase 3β), S6K (Ribosomal protein S6 kinase), DYRK1B (Dual-specificity tyrosine phosphorylation regulated kinase 1B) that can affect the condition of Gli1/2 phosphorylase, thus regulating its activity [126,127,128]. DYRK1B and S6K are also thought to be associated with the mTOR (mammalian target of rapamycin)/Akt (Protein kinase B) signaling processes, where DYRK1B regulates mTOR/Akt while mTORC1 phosphorylates S6K. Therefore it appears to be evident that there is a connection between Gli1/2 and Phosphoinositide 3-kinase (P13K) mTOR/Akt. Gli plays an important role in dual regulation. Hence, if there is an over-activation of mTOR signaling because of the influence of upstream regulation causing greater S6K activity, hedgehog signaling in the non-canonical pathway becomes upregulated. The biochemical and genetic biomarkers along with oxidative stress and the BDNF biomarker measured in serum, cerebrospinal fluid (CSF), and urine, have been suggested as potential biomarkers for ASD [129]. Some more research studies are required to check the accuracy and reliability of the hedgehog pathway-related tests in identifying ASD phenotypes. Some other studies investigated the role of hedgehog pathways in phenotypic acquisition and T-cell differentiation. Considering this as a reference, the T helper and CD4+ cells, along with high levels of Gli2A, were further differentiated into Th2 cells and secreted six-fold more interleukin 4 cells (IL-4) with normal levels of Gli2A after stimulation, suggesting that IL-4, like Gli, acts as a transcriptional target [130]. It has been reported that the blood serum profile of mothers of autistic offspring show elevated levels of interleukins (IL-4) and dysregulation of T-helper cells and the lymphocytes that regulate them [131] and that autistic patients [132] have increased levels of Sonic hedgehog (Shh) and Indian hedgehog (Ihh) ligands [125]. Conclusively, Gli factors play an important role in cell growth, differentiation, and survival in both the brain and immune system. Hence, more studies are required to prove the accuracy and reliability of the hedgehog-related pathway involvement in the detection of ASD phenotypes as well as modulators for designing novel therapeutic drug targets to treat ASD.

### 4.3. Retinoic Acid (RA) Signaling Pathway

**Figure 3:**
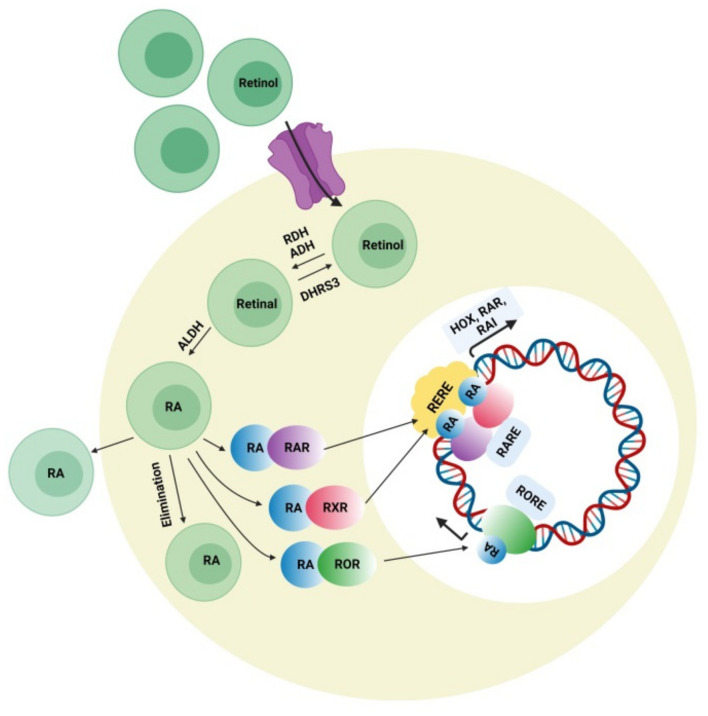
Retinoic acid (RA) signaling pathway. Retinoic acid is synthesized intracellularly from retinol, which first is converted into retinaldehyde by the enzyme alcohol dehydrogenase or retinol dehydrogenase. The reversible conversion of retinal to retinol is mediated by the enzyme retinal reductase (DHRS3). Retinal is irreversibly oxidized to become retinoic acid (RA) by the enzyme retinaldehyde dehydrogenase (ALDHs). Retinoic acid inside the cell binds to receptors present on the surface of the nucleus (RAR, ROR, and RXR) and recognizes consistent response elements (RARE, RORE, and RXRE) along with the DNA, which activates the expression of different target genes.

Retinoic acid can affect various developmental genes that contain retinoic acid response elements (RARE) together with their regulatory spaces. This role of RA suggests interconnections among neurodevelopmental disorders and RA signaling pathways. During embryonic development, retinoic acid helps regulate a set of HOX (homeobox) genes that, during embryogenesis, shapes the upper-body pattern both anteriorly and posteriorly and is involved in brain patterning. RA is engaged in neural-cell differentiation involving dopaminergic and GABAergic neurons. It is also a key induction component (together with sonic hedgehog) for the differentiation of motor neurons from pluripotent stem cells [133]. Moreover retinoic acid is also important for the normal functioning of motor neurons. It was also reported that RA is important for neural migration and neurogenesis in the granular zone of the hippocampus, sub-ventricular zone, and olfactory bulb [134]. Retinol concentration in adequate amount is required for normal functioning of RA signaling pathway presuming that all the enzymes related to RA pathway and nuclear factors work accordingly. Figure 3 represents the synthesis of retinoic acid and the retinoic acid signaling pathway. The deficiency of retinol is one of the major causes of decreased intracellular RA signaling. Some studies demonstrated that the deficiency of retinol in rats during pregnancy decreases RA receptor expression (RAR, beta isoform) in the hypothalamus, causing autistic-like symptoms in the neonates [135]. Interestingly, a lower level of retinol was detected in some autistic subjects when compared with the normal control group in China, which was possibly a synergistic factor in ASD symptom development [136]. Retinol supplements can activate RAR expression and lessen ASD symptoms. The important metabolic step in the RA pathway is the conversion of retinal to retinoic acid with the help of the enzyme (ALDH) retinaldehyde dehydrogenase, which ensures the concentration of RA in the cell. An increase in the degradation rate of this enzyme’s isoform ALDH 1A2 because of over-ubiquitinoylation by the enzyme UBE3A (ubiquitin ligase E3) was established in vitro, and autistic features were observed in mice with an overexpression of UBE3A [137]. The loss of function of UBE3A is related to a neurodevelopmental disorder (Angelman syndrome) showing its importance in brain development [138]. The nuclear receptors for RA have also been involved in ASD pathology. RORs (retinoic acid related orphan receptors) activate transcription of several genes by acting as transcriptional regulators upon retinoic acid-binding. A reduction in ROR gene expression due to hypermethylation was found in autistic subjects. In addition, an immunohistochemical analysis of the postmortem brains of autistic subjects confirmed a lower level of ROR alpha protein [139]. Disruption of the retinoic acid enzymatic production pathway was found to be associated with ASD phenotypes and retinoic acid nuclear receptors, which have also been involved in the pathophysiology of ASD; hence, additional studies have to be performed to establish the correlation between ASD pathogenesis and the involvement of RAR and ROR agonists for autism treatment. Quantitative EEG analysis is another signal-detection tool for diagnosing ASD. The details of the individual characterization of EEG fluctuations in ASD subjects could help examine issues of the brain, which would be useful for observing automatic groupings and random draws of the patient population when analyzing the sensory-processing issues of the brain and the peripheral system [140].

## 5. Future Perspectives and Conclusions

Existing knowledge and understanding related to the etiology and pathology of ASD is still growing through various collaborative and comprehensive efforts. We have to identify the additional ASD-puzzled pathologies linked with our current knowledge to develop a clear picture of ASD. A multiple domain expert collaboration is effectively required to analyze its growing genetic, epidemiological, and environmental aspects. Dysregulation of multiple signaling neurodevelopmental pathways like Wnt, Hedgehog, and RA during embryogenesis seems to cause ASD and disrupt neurogenesis. Investigation of the pathway modulators, transcriptional agents such as β-catenin and related cofactors like BCL9 (B-cell CLL/lymphoma 9 transcription co-activator), and the mechanism of the T-cell factor in the Wnt signaling pathway may prove to be beneficial in the treatment of ASD subjects. Gli factors play an important role in cell growth, differentiation, and survival in both the brain and immune system. More studies are required to prove the accuracy and reliability of hedgehog-related pathway involvement in the detection of ASD phenotypes as well as the modulators for designing novel therapeutic drug targets. Disruption of the retinoic acid enzymatic production pathway is found to be associated with ASD phenotypes, and retinoic acid nuclear receptors have also been involved in the pathophysiology of ASD; hence, additional studies have to be performed to establish the correlation between ASD pathogenesis and the involvement of RAR and ROR agonists for autism treatment. These studies will be of significant relevance to future pharmacological therapeutic interventions and the customized design of ASD modifying drugs. Systematic investigation studies are required to determine which of these signaling pathways has the most significant impact on brain function and structure, causing behavior impairment. The design of oxidative biomarkers along with ASD biomarkers might result as an important tool for diagnosing ASD symptoms. Markers for body fluids and peripheral tissue quantification are found to be suitable candidates as they represent the invasive diagnostic method. Early detection is required for an effective treatment that addresses the symptoms and permits the potential reversion to the normal conditions of neurons. Hence, more research is still required to understand the mechanistic scenario of signaling pathways, communication in ASD, and the causal gene interaction with these pathways.

## Figures and Tables

**Table 1:** Three severity levels of ASD (modified from American Psychiatric Association, 2013) [13].

Levels	Clinical Symptoms		
		Social Communication	Repetitive Behavior
Level 1	Requires extensive medical support	Severe impairment in verbal and non-verbal communication; a deficit in social interactions; less response to social overtures (e.g., rarely starts an interaction if they have some words of intelligible speech and respond only to direct social overtures.	Rigid behavior; extreme problems coping with change; repetitive or restricted behavior marked by interferences in body functioning in all spheres; great difficulty changing action or focus.
Level 2	Requires medical support	Marked impairment in verbal and non-verbal communication; deficit in social interaction even with support; minimum responses to social overtures like simple spoken sentences; less interest in interaction, and odd behavior in non-verbal communication	Rigid behavior; difficulty coping with change, repetitive or restricted behavior affecting various functions in different contexts; trouble changing action or focus.
Level 3	Requires support	Noticeable impairments in social interaction without support; problems initiating interactions with people and appears to have less interest in social communication (e.g., affected person speaks full sentences with others but to-and-fro conversation fails and attempts to make friends typically not successful and odd),	Rigid behavior causes difficulty with functioning in several contexts; problems switching from one activity to another; deficit in behavior while organizing and planning inhibits independence.

**Table 2:** List of genes implicated in ASD and ID risk.

S. No.	Genes implicated in ASD and ID	Functions and Effects	References
1.	Copy number variants (CNV) Variation in genes like *NRXNI* (neurexin 1 α), *CNTNAP2* (contactin-associated protein-like), *NLGN4* (neuroligin), *SHANK1, SHANK3* (SH3 and multiple ankyrin repeat domains 3)	Neuronal and synaptic functions: -*NRXNI*, *NLGN4*, and *CNTNAP2* causes synaptic adhesion -*SHANK* performs scaffoldings of postsynaptic density protein and the formation and maturation of dendritic spines.	[42,43,44,45,46]
2.	*OPHN1* (oligophrenin-1), *MEGAP* and *SRGAP3* (SLIT-ROBO Rho-GTPase activating gene), *OCRL1* (oculocerebrorenal syndrome of Lowe), *ARHGEF6* (Rac/Cdc42 Guanine Nucleotide Exchange Factor 6), *ARHGEF9* (Rac/Cdc42 Guanine Nucleotide Exchange Factor 9), *FGD1* (encodes guanine nucleotide exchange factor which activates Rho GTPase Cdc42), *LIMK1* (LIM domain kinase 1 regulator of actin dynamics), *PAK3* (p21-activated kinase), and *IQSEC2* (IQ (aa 347–376) and the SEC7 domains)	-Functions as effectors or regulators of Rho GTPases or Rac and Cdc42 and code for proteins linked with GTPase signaling and -Perform an important role in synaptic transmission, neurite outgrowth, and differentiation, dendrites branching, spine maintenance, and formation.	[47,48]
3.	*PAK3*	PAK3, protein, is associated with the p21-activating kinases (PAK) family, they are downstream effectors for Rac and Cdc42. Its downregulation causes spine abnormalities and defects in synaptic plasticity.	[49,50,51]
4.	*FMR1 FMRP*: Fragile X mental retardation protein	*FMR1* acts as a translational repressor show 15–30% rate of autism	[52]
5.	TSC1/2 (Tuberous sclerosis protein 1 and 2)	Gene suppressor and inhibitor of mTOR and shows 25–60% rate of autism.	[53,54]
6.	*PTEN* (Phosphatase and tensin homolog)	Gene suppressor and inhibitor of P13K and mTOR.	[53,54]
7.	*NF1* (Neurofibromin)	Gene suppressor—an inhibitor of PI3K/mTOR signaling	[53,54]
8.	*MECP2* (Methyl-CpG-binding protein-2)	Global transcriptional repressor and 100% rate of autism causes Retts syndrome	[53]
9.	UBE3A (E6AP ubiquitin-protein ligase)	Ubiquitination, 40% rate of autism causes Angelman’s syndrome	[53]
10.	*CACNA1C* (Alpha-1 subunit of a voltage-dependent calcium channel)	L-type voltage-gated calcium channels, 60% rate of autism causes Timothy syndrome	[53,55]
11.	*CTNND2* (Adhesive junction-associated δ-catenin protein)	Dendritic morphogenesis, histone modification, and participation in WNT signaling	[53]
